# Tumor response and tolerability under fractionated x-ray irradiation in a mouse xenograft model

**DOI:** 10.1088/1361-6560/ae7953

**Published:** 2026-06-25

**Authors:** Kailey Choi, Jenny Szu, Juan Antonio Camara Serrano, Veronica Steri, Chad Gunther, Youngho Seo, Robin Peter

**Affiliations:** 1 Physics Research Laboratory, Department of Radiology and Biomedical Imaging, University of California, San Francisco, California, United States of America; 2 Department of Neuroscience, University of California, Berkeley, California, United States of America; 3 Helen Diller Family Comprehensive Cancer Center, University of California, San Francisco, California, United States of America; 4 C&C Irradiator Service, LLC, Washington, DC, United States of America; 5 Department of Nuclear Engineering, University of California, Berkeley, California, United States of America; 6 Department of Radiation Oncology, University of California, San Francisco, California, United States of America

**Keywords:** x-ray irradiator, *in vivo* tumor model, fractionated dosing, quality control, dosimetry

## Abstract

*Objective.* Modern self-contained x-ray irradiators offer a safer alternative to radionuclide sources for preclinical radiation research and can deliver conformal, clinically relevant dose distributions. However, concerns persist regarding their performance and biological toxicity as direct replacements in radiobiological studies. We assessed two preclinical x-ray platforms, a collimated cabinet X-Rad320 and an image-guided small animal radiation research platform (SARRP), for induced tumor response, tolerability, and dosimetric accuracy in a subcutaneous xenograft prostate tumor model under 2 Gy daily fractionated radiotherapy. *Approach.* Male athymic mice bearing 22Rv1 xenografts were randomized into cohorts receiving total doses of 10, 16, or 20 Gy (5, 8, or 10 2-Gy fractions) on either platform. Unirradiated controls were included. Tumor burden was quantified by the area under the tumor growth curve (AUC). Machine dose delivery accuracy was verified quarterly with alanine pellet dosimetry. *Main results.* Across regimens, no animals met humane body condition or weight endpoints or exhibited overt clinical toxicity. All irradiated groups had lower tumor normalized AUCs than controls, but the dose response was not clearly monotonic, and not all differences remained statistically significant after correcting for multiple comparisons. At 14 d post-treatment, SARRP at 16 Gy provided the greatest tumor suppression, with no significant additional benefit observed with 20 Gy. Histological *γ*-H2AX staining provided complementary evidence of a radiation-induced dose response. *Significance.* Focused x-ray platforms can reproducibly deliver conventional fractionated radiotherapy regimens in mice, inducing tumor response without overt clinical toxicity. However, platform-specific dose deviations highlight the need for rigorous dosimetric calibration and quality assurance to ensure they can effectively replace cesium-137 for *in vivo* research with comparable tumor control outcomes.

## 1. Introduction

The use of cesium-137 (^137^Cs) for research irradiation poses security, safety, and regulatory challenges, including the potential use in a radiological dispersal device if lost or stolen. In 2025, the U.S. National Nuclear Security Administration’s (NNSA) Office of Radiological Security removed 68 high‐activity devices as part of its plan to eliminate cesium-based blood sterilization by 2027 (National Nuclear Security Administration 2026). This effort is part of the broader Cesium Irradiator Replacement Project (CIRP), which aims to replace ^137^Cs devices in medical and research settings with non-radioisotopic, electrically generated x‐ray irradiators (National Research Council 2008). Comparative studies have shown that advanced small‐animal x‐ray irradiators can provide biological effects comparable to those of ^137^Cs, though their spectral differences introduce some discrepancies depending on target geometry and material (Murphy and Kamen 2019, Poirier *et al* 2020, Caravaca *et al* 2022). Additionally, compared to aperture-based ^137^Cs systems without customizable collimation (Fontanarosa *et al* 2020), modern small‐animal x‐ray irradiators offer more flexibility for conforming dose distributions to limit damage to healthy tissue. Two common platforms include the cabinet irradiator X‐Rad320 (Precision X-Ray, North Branford, CT, USA), which uses a 320 kVp x‐ray tube with adjustable collimator leaves to produce rectangular fields, and the small animal radiation research platform (SARRP, Xstrahl Life Sciences, Suwanee, GA, USA), which integrates cone‐beam micro‐CT (*µ*CT), computer‐controlled motion stages, and 3D treatment planning to reproduce a clinical workflow in rodents.

Laboratories are already using x-ray platforms for clinically adjacent therapy studies, but concerns still exist about increased toxicity associated with x-ray irradiators (Gibson *et al* 2015, Bell *et al* 2022, Keam *et al* 2022). Additionally, inadequate quality assurance (QA), quality control (QC), and reporting in preclinical irradiation has resulted in documented deviations between intended and delivered dose, obscuring the interpretation of many findings (Pedersen *et al* 2016, Biglin *et al* 2022, Hill *et al* 2025). There is therefore a need for reproducible preclinical studies, using calibrated or dosimetry-informed machine outputs with reported machine settings, to assess the tolerability and tumor effect achievable with preclinical x-ray irradiators.

A previously developed QA protocol utilizes alanine pellet dosimeters in 3D printed mouse phantoms, together with Monte Carlo dose simulations, to convert nominal x-ray settings into the expected absorbed dose in tissue (Caravaca *et al* 2022, Duncan and Gunther 2025). This approach provides traceable measurements and detailed dose distributions, supporting reproducible reporting. In the present study, the output validation QC component of the QA program is applied to compare two collimated x‐ray platforms and to quantify platform‐specific dose deviations.

Using this dosimetry-validated framework, we investigated how absorbed dose influences growth of 22Rv1 prostate xenografts *in vivo* under fractionated x‐ray irradiation following a conventional clinical regimen of 2-Gy daily fractions, five days per week, over several weeks. 22Rv1 is widely used as a preclinical model for castration-resistant prostate cancer to investigate the mechanisms behind resistance to standard first-line treatments including radiation, docetaxel and anti-androgen therapy (see supplementary information). In this study, male athymic mice bearing subcutaneous 22Rv1 prostate xenografts were randomized into cohorts receiving 10, 16 or 20 Gy total doses (5, 8, or 10 2-Gy fractions) on either a collimated cabinet X‐Rad320 or an image‐guided SARRP; an unirradiated control cohort was included. Tumor response was assessed by calculating the area under the tumor growth curve and histological DNA damage markers in harvested tumor tissue, and tolerability was monitored using body‐weight change. We hypothesized that both platforms would achieve tumor growth suppression with acceptable toxicity, but that differences in dose delivery and spatial targeting might lead to differences in tumor control. These results support ongoing efforts to replace high‐activity gamma sources with safer x‐ray technologies and provide insight into the dosimetric and biological factors that must be addressed for successful translation of preclinical irradiation studies.

## 2. Methods and materials

### 2.1. Experimental design

The animal experiments were approved by and carried out in compliance with the Institutional Animal Care and Use Committee (IACUC) and established guidelines at the Laboratory Animal Resource Center (LARC) at the University of California, San Francisco under IACUC protocol no. AN206976.

Male athymic mice were inoculated subcutaneously with 22Rv1 prostate‐cancer cells. Because xenograft growth rates varied widely, animals were enrolled on a rolling basis once their tumors reached 400–500 mm^3^, ensuring comparable baseline volumes across cohorts. Enrolled mice were randomized into six irradiated cohorts (*n* = 5 per cohort) and an unirradiated control cohort, the latter reduced to five animals by omitting the largest and smallest tumors to match sample sizes. Total doses of 10, 16 and 20 Gy were administered as 2-Gy fractions: the 10 Gy regimen comprised five consecutive daily fractions, the 16 Gy regimen eight fractions, and the 20 Gy regimen ten fractions.

Tumor dimensions and body weight were recorded twice weekly beginning on the first fraction (day 1) through the standardized endpoint, 14 d after the end of the last fraction (19–27 d from treatment initiation). Tumor volume analyses, body-weight data, and histological endpoints (*γ*‐H2AX staining) were evaluated at the standardized endpoint. Tumor dimensions were measured with digital calipers, then tumor volumes (*V*, mm^3^) were calculated using the standard ellipsoid‐approximation formula *V* = (length × width^2^) × 0.52 (Puskás *et al* 2024). Irradiated mice were anesthetized with isoflurane for each fraction, whereas control animals were handled similarly but did not receive anesthesia or radiation. Blinding was not implemented during data collection, as tumor measurements were performed with standard procedures.

### 2.2. Irradiation platforms

Two x-ray irradiator systems were used in this study: a cabinet-style biological irradiator (X-Rad320) and an image-guided SARRP. In each case, animals were anesthetized with isoflurane and positioned in a reproducible ventral recumbency configuration with the tumor positioned towards the field. The mice were not otherwise immobilized during the process.

The X-Rad320 system was used to deliver x-ray irradiation with a rectangular field collimated by four motorized adjustable leaves. Irradiations were performed at a source-to-platform distance of 60 cm at 320 kVp and 12.5 mA over 200 s (1 cGy s^−1^) using vendor filter no.2 (1.5 mm Al, 0.25 mm Cu, and 0.75 mm Sn). 2 Gy treatment fractions were delivered to each five-mouse cohort simultaneously, with the field size selected to cover all tumors with a small margin. The positioning of the subjects within the field was verified with the field light (supplementary figure S1).

The SARRP system integrates on-board cone-beam micro-computed tomography (*µ*CT) with x-ray beam delivery to enable image-guided irradiation. For SARRP treatments, animals were imaged immediately prior to irradiation using *µ*CT to verify positioning. Tumor location was identified on the acquired images to select the treatment target (supplementary figure S2). Treatment planning system (TPS) isocenter selection accounted for differences in the source-to-surface distance (SSD) from the calibration SSD of 35 cm caused by subject posture. Vendor specifications report targeting accuracy of up to 0.2 mm and 5% or better dosimetric agreement between calculated and delivered beam penumbra for field sizes under 5 mm. 2 Gy treatment fractions were then delivered using static collimated (1–5 mm) x-ray beams according to the standard *µ*CT planning workflow with beam parameters of 220 kVp, 13 mA and 0.15 mm Cu filtration over approximately 70 s (2.9 cGy s^−1^).

Because animals were enrolled on a rolling basis as tumors reached the target size, the calendar timing of fraction delivery differed between dose cohorts (supplementary figure S3). Weekend breaks were incorporated when applicable, consistent with standard fractionated radiotherapy practice. All cohorts received the same nominal fraction size (2 Gy) and completed their prescribed total dose.

### 2.3. QC measurements

A previously validated QC protocol was performed to verify the consistency and stability of the x-ray irradiators used in this study (Caravaca *et al* 2022, Duncan and Gunther 2025). For each irradiator, 3D-printed rodent biophantoms containing alanine pellets were irradiated using the same nominal beam settings and collimation configurations employed during animal treatments. The absorbed doses delivered to the pellets were measured by NIST through their electron paramagnetic resonance (EPR) protocol. A published proprietary correction factor was applied to account for dosimetric differences in alanine in response to orthovoltage x-rays versus Co-60, the NIST reference standard for EPR dosimetry (Duncan and Gunther 2025). QC was conducted quarterly over the year leading up to the irradiation study to verify the output consistency of the machines. In this study, nominal dose refers to the user-defined machine prescription, and absorbed or delivered dose is reported as the measured quantity from NIST-traceable alanine dosimetry.

### 2.4. Animal model and xenograft implantation

Forty male mice (8–10 weeks old) were purchased from Taconic Biosciences (model no. NCRNU-M; NCr-*Foxn1^nu^*) and housed with ad libitum food and water in a 12 h-light cycle within the animal facility of the UCSF Helen Diller Comprehensive Cancer Center Research Building. Using 1.5 × 10^6^ 22Rv1 prostate cancer cells suspended in serum-free medium and Matrigel (1:1), each mouse was subcutaneously inoculated on the right flank (100 *µ*l per injection).

### 2.5. Tumor volume analysis

Longitudinal tumor burden was summarized using the area under the tumor growth curve (AUC) from treatment initiation until the standardized endpoint. AUCs were computed by trapezoidal integration between successive measurements. For each mouse, tumor volumes *V_k_ (*measured on day *t_k_)* and at the next measurement *V_k+1_ (*measured on day *t_k+1_)* were connected by straight‐line segments. The area under the curve between two successive time points was approximated by the trapezoidal rule as $\left[ {\left( {{V_k} + {V_{k + 1}}} \right)/2} \right]*\left( {{t_{k + 1}} - {t_k}} \right).$ Summing these trapezoids across all time intervals yielded the total AUC for each animal.

Because mice were enrolled and treated on different dates and over courses of varying length, the observation duration varied slightly across animals. To standardize comparisons, we normalized each mouse’s AUC by dividing by its individual follow‐up interval, (*t*_last_–*t*_first_). The resulting normalized AUC (nAUC), expressed in mm^3^·d^−1^, represents the average tumor burden per day and condenses each growth trajectory into a single scalar metric. AUC-based metrics have been advocated as a useful tool for comparing tumor growth kinetics because they reflect the entire curve and can be restricted to defined periods (Duan *et al* 2012). By calculating the nAUC and its standard deviation (SD) for each cohort, average tumor burdens and their respective relative uncertainties were calculated.

To test whether irradiation reduced tumor burden relative to controls, two‐sample Welch’s *t*‐tests compared each treatment cohort’s nAUC with that of the control cohort. To control the family-wise error rate across six cohort comparisons, *p*-values were adjusted using the Holm–Bonferroni method (Holm 1979, Aickin and Gensler 1996). Mean differences in nAUC relative to control, 95% confidence intervals, *p*‐values and Holm–Bonferroni-adjusted *p*‐values were calculated. Two-sample Welch’s *t*-tests were also conducted to compare cohorts treated to the same nominal dose between platforms. Analyses assumed normal distribution of mouse-level AUC within each cohort and independence between animals. Mixed-effects modeling was not used because the primary tumor-growth endpoint was the normalized AUC, which summarizes each animal’s longitudinal tumor response into a single interpretable scalar outcome rather than modeling repeated tumor-volume measurements over time. The durability of response and post-treatment regrowth were evaluated qualitatively from tumor growth trajectories.

### 2.6. Statistical analysis of body weight

Body weight was assessed as a measure of tolerability rather than a primary efficacy endpoint. In accordance with institutional IACUC guidelines, a significant loss of body mass was defined as a reduction of ⩾ 15% of baseline body weight between day 1 and the standardized endpoint. Baseline body weight (BW_1_) was recorded on day 1 for each mouse and compared to body weight at the standardized endpoint (BW*_f_*). The raw percentage change in body weight for each mouse was calculated as $\% \Delta {\mathrm{B}}{{\mathrm{W}}_{{\mathrm{raw}}}} = \left( {{\mathrm{B}}{{\mathrm{W}}_f} - {\mathrm{B}}{{\mathrm{W}}_1}} \right)/{\mathrm{B}}{{\mathrm{W}}_1}*100$.

For each five‐mouse cohort, we computed the mean percentage change, SD and range (minimum–maximum). To evaluate treatment‐related effects, two‐sample Welch’s *t*‐tests compared each irradiated cohort with the control cohort, and exploratory *t*‐tests compared X‐Rad320 and SARRP cohorts at the same nominal dose. *P*‐values were adjusted for multiple comparisons using the Holm–Bonferroni method (family‐wise *α* = 0.05). No additional modeling was applied because body weight was treated as a tolerability endpoint.

Because tumor mass contributes materially to measured body weight and differs across groups, we also calculated a tumor‐mass-corrected (net) body‐weight change for each mouse. Final tumor mass at the standardized endpoint (*M*_tumor,*f*_) was estimated by converting the measured tumor volume to mass using an assumed density of 1 mg·mm^−3^ (i.e. 1 mm^3^ ≈ 1 mg). For each irradiated mouse, we computed a tumor‐mass correction term equal to the difference between that mouse’s estimated final tumor mass and the control cohort’s mean final tumor mass $(\mathop M\limits^ - $_control tumor_, *_f_*). Net percentage change in body weight was then calculated as $\% \Delta {\mathrm{B}}{{\mathrm{W}}_{{\mathrm{net}}}} = \left[ {\left( {{\mathrm{B}}{{\mathrm{W}}_f} - {\mathrm{B}}{{\mathrm{W}}_1}} \right) - \left( {{M_{{\mathrm{tumor}},f}} - {{\bar M}_{{\text{control tumor}},f}}} \right)} \right]/{\mathrm{B}}{{\mathrm{W}}_1}*100$, where a negative value indicates weight loss. This adjustment isolates systemic weight change by removing the component attributable to having a larger or smaller tumor than the control average at the endpoint.

The study design also accounted for potential confounders. Control animals were handled without anesthesia, whereas irradiated animals were anesthetized for each fraction, so weight comparisons were interpreted cautiously. In addition to quantitative weight measurements, mice were monitored for clinical signs of radiation‐induced toxicity (e.g. decreased body condition score, reduced motility, gastrointestinal side effects).

### 2.7. Histological analysis

The *γ*-H2AX stain images were acquired post-treatment to compare the DNA double-strand breaks (DSBs) resulting from X-Rad320 and SARRP treatments and verify targeting to the tumor area. Following euthanasia, tumor tissues were harvested, fixed in 10% neutral buffered formalin, processed with ethanol gradient (from 30% to 70%), and paraffin-embedded. Routine histological analysis was performed to assess the radiation induced DNA damage. Tissue sections of 4 *µ*m thickness were air dried and baked (at 60 °C for two hours) prior to histological staining. Staining was performed in Ventana Discovery Ultra carousel using primary antibody for phospho-*γ*-H2AX (Ser139) (CST # 9718 S, 1:400 dilution). For detection and visualization anti-HRP/DAB based Discovery Chromomap RUO detection reagents (Ventana Cat. no. 760-4820; no. 760-159) were utilized. Sections were counterstained with Hematoxilin II and Bluing reagents (Ventana Cat, no. 790-2208; no. 760-2037). Images were captured with Zeiss Axio ScanZ.1er (20X). Samples were collected from a subset of 18 total mice, using 2–3 mice from each treatment or control group (supplementary table SI).

To quantify the *γ*-H2AX staining images, we utilized QuPath, an open-source software designed to analyze digital pathology in research (Bankhead *et al* 2017). In each sample, one representative scene with the highest *γ*-H2AX staining area was selected for analysis. A standardized region of interest was defined for each selected scene in which the percentage of *γ*-H2AX-positive cells was calculated, expressed as the percentage of positive foci per square millimeter (%foci mm^−2^).

## 3. Results

### 3.1. Dose delivery QC results from alanine pellets

**Figure 1.**
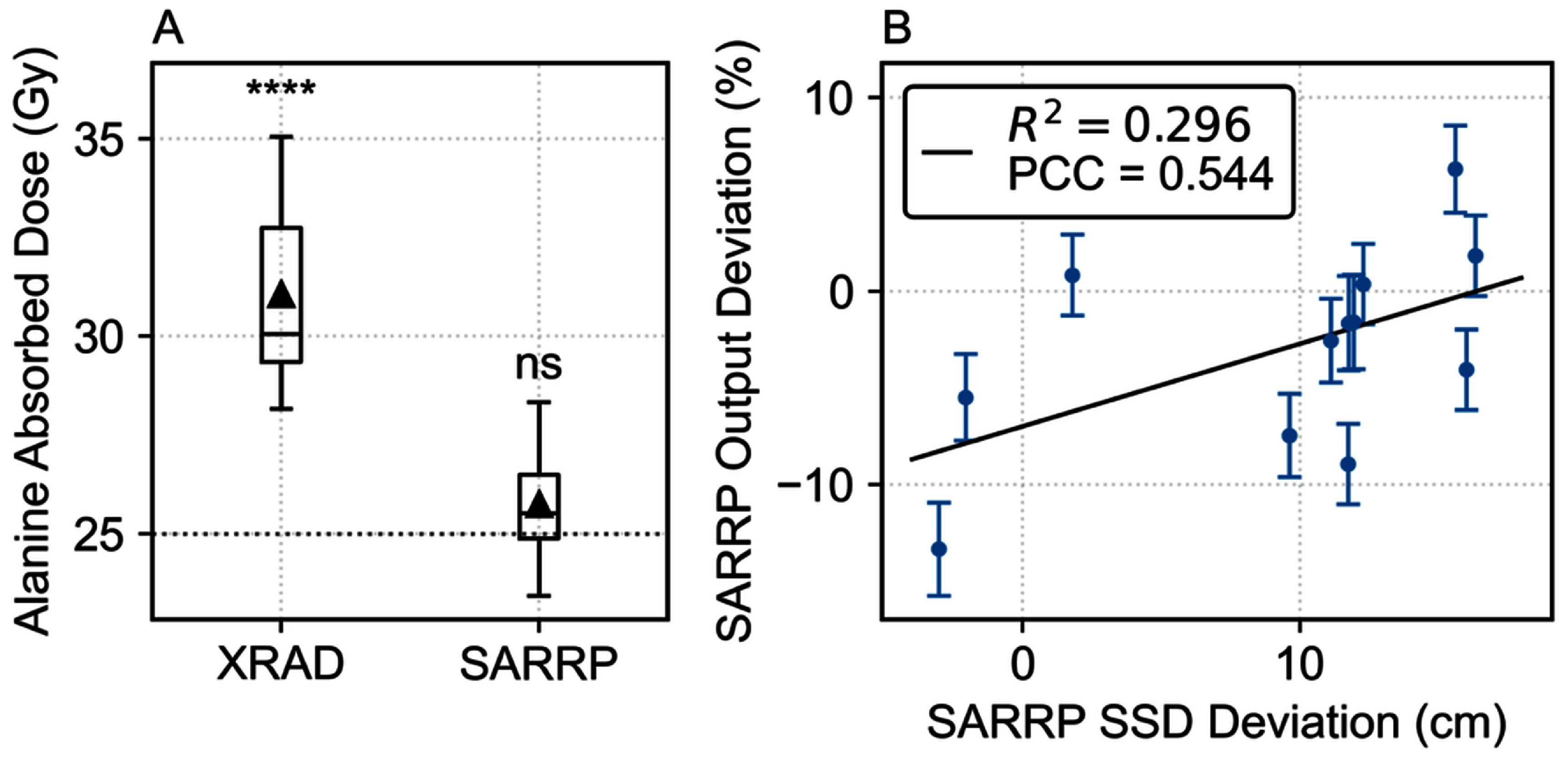
Quarterly QC measurements. (A) QC output measurements show SARRP delivery consistent with, and X-Rad320 deviation from, 25 Gy nominal dose calibration protocols. Boxplots show the −1.5 to +1.5 IQR with median denoted by the central bar and mean by the triangle (X-Rad320: *n* = 15, SARRP: *n* = 12). In one-sample *t*-test comparison to 25 Gy: ****p < 0.0001, ns = not significant. (B) Weak correlation between SARRP SSD and output deviation (Pearson’s correlation coefficient PCC = 0.544, *p* = 0.067).

Quarterly alanine‐pellet QC measurements confirmed previously observed deviation between prescribed nominal dose and delivered absorbed dose on X‐Rad320 (figure 1(A)). Under a nominal 25 Gy calibration setting, measured doses ranged from 28.2 to 35.1 Gy (*n* = 12), with a mean (±SD) of 31.1 ± 2.4 Gy (24.3 ± 9.5% deviation). In a one-sample *t*-test comparison to 25 Gy, the *t*-statistic was 9.9 (*p* = 10^−7^). SARRP measurements (*n* = 12) were generally closer to prescription and showed reduced variability (figure 1(A)). Setup variation produced SSD differences from calibration isocenter at 35 cm (9.4 ± 6.8 cm: range −3.0–16.4 cm), but absorbed doses were not significantly different from nominal, with a mean of 25.7 ± 1.3 Gy (3.0 ± 5.3% deviation). Still, weak correlation between SSD deviation and output deviation (Pearson’s coefficient: 0.544, *p* = 0.067) suggested that the SARRP TPS may not completely model all beam delivery aspects (figure 1(B)). No SSD comparison was made for X-Rad320, since the system only considers stage height and does not report the actual geometric SSD.

### 3.2. Tumor volume progression

**Figure 2.**
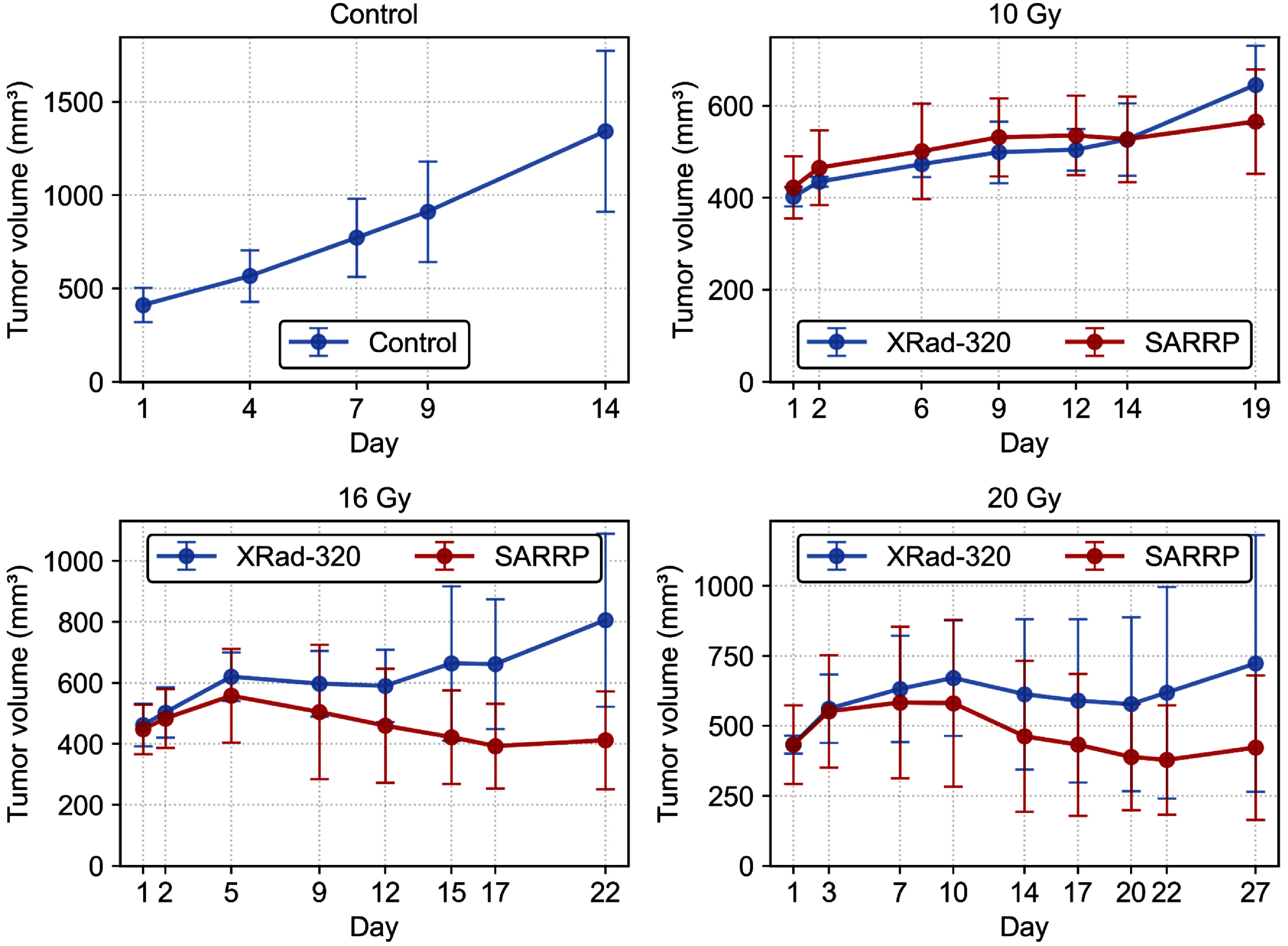
Tumor volume curves. Tumor volume curves for unirradiated control, 10 Gy, 16 Gy, and 20 Gy cohorts (*n* = 5 per cohort) show growth suppression relative to control. Mean and standard deviation shown with error bars.

Untreated control tumors grew rapidly and heterogeneously, whereas all irradiated groups (*n* = 5 each) showed suppressed volume curves (figure 2). At 10 Gy, both X-Rad320 and SARRP delayed growth relative to control, though tumors continued to enlarge. At 16 and 20 Gy, divergence between platforms was visible: SARRP‐treated tumors exhibited sustained suppression and occasional regression, whereas X‐Rad‐treated tumors showed slowed growth without consistent regression. Intra-cohort variability was observed in the tumor volume curves of individual subjects, reflecting a mix of durable responses and partial regrowth (supplementary figure S4).

**Figure 3.**
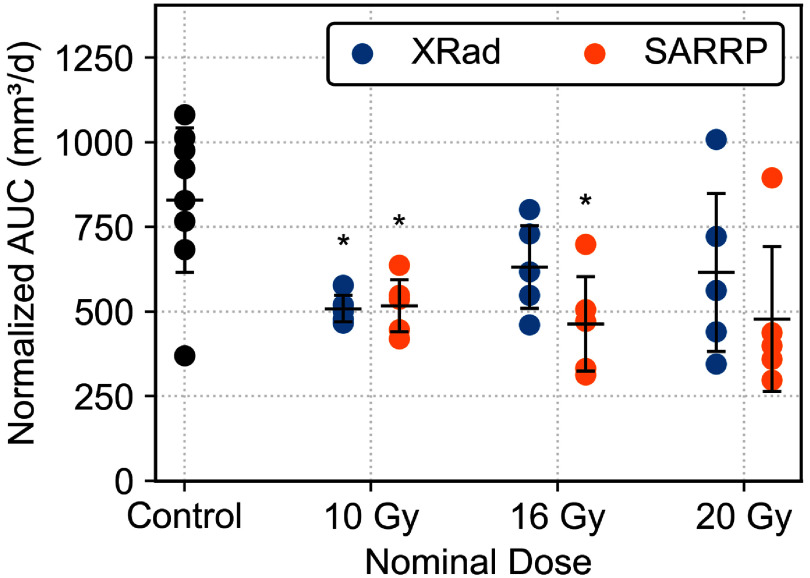
Normalized AUC by treatment group and nominal dose (*n* = 5 per cohort). Bars show mean and standard deviation. In Welch’s *t*-test comparison to control: *Holm–Bonferroni adjusted *p* < 0.05. Negative change relative values indicate lower tumor burden compared with the control.

These trends are quantified by the nAUC values summarized in table 1 and figure 3. The control group had the largest average nAUC (830 mm^3^·d^−1^). All irradiated cohorts showed lower nAUC relative to control, but dose responses were not clearly monotonic. X-Rad320 10 Gy, 16 Gy, and 20 Gy cohorts reduced mean nAUC by 39%, 24%, and 26%, respectively (*n* = 5 each). SARRP 10 Gy, 16 Gy, and 20 Gy cohorts reduced mean nAUC by 38%, 44%, and 42%, respectively (*n* = 5 each). After Holm–Bonferroni correction for multiple testing, nAUC values for the X-Rad320 10 Gy and SARRP 10 and 16 Gy cohorts were significantly different from control (adjusted *p*‐values < 0.03; table 1). The SARRP 20 Gy nAUC was significant prior to adjustment (*p* < 0.03) but not after multiple comparisons correction (adjusted *p* > 0.05). No significant differences were found in the other cohorts, or between cohorts at the same nominal dose between platforms (*p* > 0.1, adjusted *p* > 0.3).

**Table 1.** AUC comparisons to control and statistical significance (*n* = 5 per group). Mean difference in normalized AUC versus the control cohort, 95% confidence intervals, *p*‐values, and Holm–Bonferroni-adjusted *p*‐values.

Group	Mean difference vs control (mm^3^ d^−1^)	95% CI lower	95% CI upper	*p*-value	Adjusted *p*-value
X-Rad 10 Gy	−322	−514	−129	0.0050	0.0298
X-Rad 16 Gy	−199	−422	24	0.0753	0.1506
X-Rad 20 Gy	−214	−543	115	0.1703	0.1703
SARRP 10 Gy	−312	−513	−112	0.0061	0.0298
SARRP 16 Gy	−366	−601	−131	0.0057	0.0298
SARRP 20 Gy	−352	−659	−45	0.0293	0.0879

10 Gy cohorts showed lower within-cohort variability compared to cohorts treated on the same machine at higher dose. In the 20 Gy SARRP cohort, four of five animals showed strong tumor suppression, while one outlying animal demonstrated markedly higher nAUC. This animal’s initial tumor volume was nearly twice that of the other four (678 mm^3^ vs mean 372 mm^3^, supplementary figure S4(G)), which may have contributed to the difference in nAUC under identical treatment. On X-Rad320, the 20 Gy cohort showed distributed nAUC variability. A post-hoc sensitivity analysis excluding the larger-tumor animal produced a mean nAUC reduction from control of 54% (versus 42% without exclusion), suggesting that the results may reflect disproportionate influence of this single animal on within-cohort variance. Nonetheless, the overall data suggest that image‐guided SARRP at 16 Gy provided the greatest tumor control among the regimens tested, as measured by nAUC.

### 3.3. Body weight change calculations

All animals survived to the predefined standardized study endpoint (14 d after treatment delivery), and no animals met humane euthanasia criteria. No animals exhibited clinical signs of radiation-induced toxicity, such as decreased body condition score, reduced motility, or gastrointestinal side effects. Body weight changes were assessed as a coarse tolerability indicator.

Across all cohorts, raw percentage changes in body weight were modest (⩽ 11%), and no animal exceeded the humane endpoint of ⩾ 15% weight loss. Table 2 summarizes the raw and net percent change in body weight for each cohort (%ΔBW). Briefly, the control group showed a mean increase (%ΔBW_raw_) of approximately 3.4% (SD ≈ 4.2 %, range −2.4%–11.3 %), whereas irradiated groups exhibited small decreases or modest gains. For example, the X-Rad320 10 Gy cohort lost about 6% of baseline body weight on average (SD ≈ 1.2%), and the SARRP 20 Gy cohort gained roughly 1% (SD ≈ 6.2 %). Welch’s *t*‐tests comparing each irradiated cohort to controls indicated statistically significant but small differences only for the X-Rad320 10 Gy and SARRP 16 Gy groups after adjustment for multiple comparisons.

**Table 2.** Cohort-level raw and tumor-mass-corrected (net) percent change in body weight from day 1 to the standardized endpoint (*n* = 5 per group). %ΔBW_raw_ is expressed as mean ± SD (min, max). Negative values indicate weight loss. No mouse exceeded the 15% weight-loss threshold.

Group	%ΔBW_raw_	*t*-statistic	*p*-value	Adjusted *p*-value	%ΔBW_net_
Control	3.4 ± 4.2 (−2.4, 11.3)				
X-Rad 10 Gy	−6.0 ± 1.2 (−7.2, −4.1)	−6.2	0.0001	0.0009	−5.5 ± 1.2
X-Rad 16 Gy	−3.9 ± 3.4 (7.7, 1.6)	−3.51	0.0056	0.0334	−2.9 ± 3.4
X-Rad 20 Gy	−0.8 ± 5.7 (−10.9, 2.1)	−1.46	0.1897	0.9487	0.5 ± 5.7
SARRP 10 Gy	−4.5 ± 3.0 (−7.4, 0.4)	−4.03	0.002	0.014	−4.2 ± 3.0
SARRP 16 Gy	−3.9 ± 2.3 (−5.8, −0.3)	−4.23	0.0012	0.0093	−3.1 ± 2.3
SARRP 20 Gy	1.1 ± 6.2 (−9.2, 7.6)	−0.73	0.4947	1.4841	2.3 ± 6.2

Adjusting for estimated tumor mass reduced the apparent magnitude of weight changes (%ΔBW_net_). After correction, the largest weight change was a net loss of about 5.5% in the X-Rad320 10 Gy cohort, and some cohorts (e.g. X-Rad320 20 Gy) showed little or no net weight change (mean ∼0.5%). These net changes were uniformly smaller than their corresponding raw values, supporting the interpretation that most of the apparent ‘weight loss’ in treated cohorts reflects reduced tumor burden rather than systemic toxicity. The absence of clinical radiation toxicity signs, combined with the modest magnitude of weight changes, supports the overall tolerability of the fractionated regimens.

### 3.4. DNA damage response

**Figure 4.**
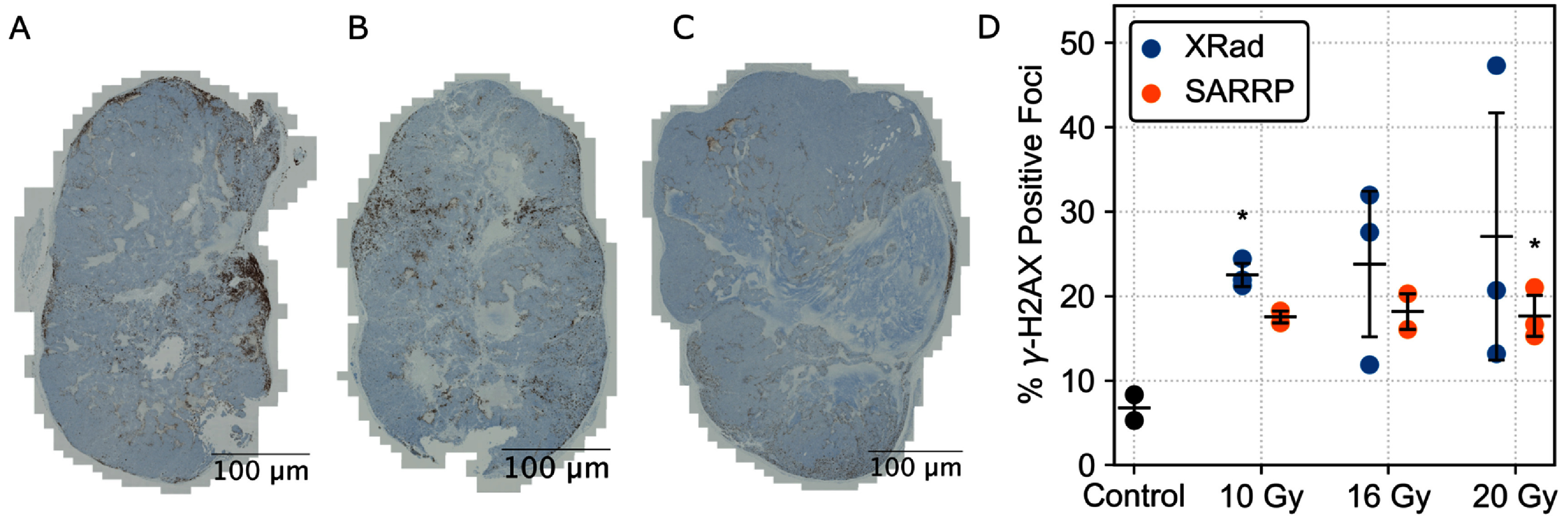
*γ*-H2AX foci stain analysis. (A) Sample exposed to 16 Gy with X-Rad320. (B) Sample exposed to 16 Gy with SARRP. (C) Non-irradiated control sample. (D) Percentage positive foci analysis across x-ray irradiation doses and platforms (*n* = 18 total). Bars show mean and standard deviation. In Welch’s *t*-test comparison to control: *p < 0.05. Both X-Rad320 and SARRP systems induced DNA damage, but higher variance was observed in X-Rad320 at all doses.

Figure 4 shows representative images of *γ-*H2AX staining in samples exposed to 16 Gy irradiation delivered using X-Rad320 (A) or SARRP (B), compared with non-irradiated control samples (C). The percentage of *γ-*H2AX-positive foci across different doses of X-Rad320 and SARRP, based on data from 6 groups of mice (*n* = 18 total), is shown in (D). Individual data points are shown; bars illustrate the mean and SD in each group.

Under X-Rad320 treatment, the mean percentage of *γ-*H2AX-positive foci were 22.0% (10 Gy), 27.6% (16 Gy), or 20.7% (20 Gy). In the SARRP group, the mean percentages were 17.6% (10 Gy), 18.2% (16 Gy), or 16.7% (20 Gy). The control (unassigned) group showed 6.8% mean *γ-*H2AX-positive foci. Within 10 Gy groups, SDs were within 3%, but the 16 Gy X-Rad320 group and 20 Gy SARRP group both showed higher variance, with 10.6% and 18.0% SDs, respectively.

Welch’s *t*-tests were conducted to compare dose groups with the control and between X-Rad320 and SARRP at equivalent doses. The 10 Gy X-Rad320 and 20 Gy SARRP groups showed significantly higher mean values than the control group (*t*(2.4) = 8.06, *p* = 0.015; *t*(2.9) = 5.15, *p* = 0.015, respectively). The 10 Gy X-Rad320 group was also significantly higher than the 10 Gy SARRP group (*t*(2.8) = 3.91, *p*= 0.03). Other comparisons did not reach statistical significance, likely due to the small sample size (*n* = 2–3 per group). Cohen’s *d*-values ranged from 0.75 to 8.24, suggesting large to very large effects.

## 4. Discussion

Historically, research irradiators have used radioactive sources such as ^137^Cs, but these pose substantial security risks and are being phased out by programs such as the U.S. NNSA’s CIRP. Modern x‐ray platforms provide an alternative and offer conformal beam delivery and the capacity to replicate the 1.8–2 Gy per fraction schedules used in clinical radiotherapy. Our study used subcutaneous 22Rv1 prostate-cancer xenografts in athymic mice to assess dose-response and tolerability in two such platforms: a collimated cabinet irradiator (X‐Rad320) and an image‐guided SARRP.

In quarterly alanine/EPR dosimetry QC, the X‐Rad320 consistently delivered higher absorbed doses than nominal (24.3 ± 9.5% deviation), whereas SARRP doses matched prescription more closely (3.0 ± 5.3% deviation). The purpose of these QC measurements was to accurately quantify dose delivery and assess the baseline performance of each device accessible to most users, rather than to perform absolute dose calibration within this study. Use of nominal beam settings with accurate dosimetric quantification allows this study to function as an easily interpretable reference data set for other users of the same machines. The results illustrate that manufacturer calibration alone does not necessarily guarantee dosimetric accuracy. This is a known reality in clinical practice, where radiation therapy systems undergo extensive independent commissioning and QA to verify and maintain calibration accuracy (Klein *et al* 2009, Miften *et al* 2018). QA protocols suitable for preclinical irradiators have been discussed in literature and consensus documents but remain limited in practice (Ma *et al* 2001, Carpenter 2024).

Differences in the two systems’ dose calculation methods likely contributed to the discrepancies in delivered dose. In SARRP planning, the machine output for a prescribed dose is informed by a convolution-superposition dose calculation accounting for geometry and material data from the *µ*CT image. This information allows a calibrated image-based planning system to consistently deliver the specified prescription dose across a variety of patient or subject anatomies and setup positions. This study’s QC measurements provide a simple example, in which dose delivery from SARRP remained mostly accurate across SSD setup variations of up to 16.4 cm. X-Rad320 also partially accounts for setup with a correction-based output adjustment defined by platform height and monitored by an ion chamber for automatic exposure control. However, dosimetric differences between correction-based methods, as opposed to model- (e.g. convolution-superposition) or Monte Carlo-based treatment planning, are well documented academically and clinically (Reft *et al* 2003, Chen *et al* 2019). In correction-based planning, the actual absorbed dose delivered to the target is still affected by differences in geometry, materials, and backscatter relative to calibration conditions. While these systems are often able to achieve good accuracy in homogeneous media, reproducible animal experiments in cabinet systems require, at minimum, output QA and consideration of target geometry. Together, these findings underscore the need for rigorous absorbed-dose calibration protocols with geometry- and parameter-specific dose planning in preclinical irradiator studies, whether through vendor-provided systems or in-house modeling.

A key objective of this work was to assess biological outcomes under nominally matched prescriptions in the context of platform-specific dose deviations. Despite the excess dose delivery from the X-Rad320, no subjects in any cohort showed overt clinical toxicity. Tumor growth analysis of nAUC at the same nominal doses even showed larger effect sizes in SARRP, although these differences were not significant in pairwise comparisons. Both platforms induced tumor suppression in the form of significant nAUC differences from control at 10 Gy, but despite large nAUC reductions of 26% (X-Rad320) and 44% (SARRP), higher nominal dose treatments were not statistically significant. Large within-cohort variance may partially explain the lack of clear monotonic dose responses. Convergence of nAUC between 16 and 20 Gy treatments may alternatively indicate that further effects may only be observed with longer follow-up, or that escalation beyond 16 Gy offers limited additional benefit. The apparent dose response plateau may reflect a combination of radiobiological factors, including saturation in tumor cell kill, heterogeneity in xenograft radiosensitivity, or microenvironmental factors such as hypoxia or sublethal damage repair.

Histological *γ*‐H2AX staining, a surrogate for DNA DSBs, provided a complementary endpoint bridging physical dose and biological outcome. Significantly elevated *γ*‐H2AX foci, potentially consistent with the excess dose delivery on X-Rad320, were observed in the 10 Gy X-Rad320 cohort compared to SARRP at the same nominal dose. All irradiated groups showed increased *γ*‐H2AX foci relative to controls, but although the effect sizes were large, statistical significance was only reached in two cohorts. Taken together, the nAUC and *γ*‐H2AX analyses show that both x-ray platforms can induce tumor response, but they also reflect the limitations of modest cohort sizes given the high biological variability inherent in xenograft models.

Differences in beam quality and delivery technique between the systems may also complicate differences in biological response. SARRP operates at 220 kVp, whereas X-Rad320 was operated at 320 kVp. Biological effect per unit dose is generally known to increase with lower photon energy, but the exact differential may depend on beam parameters, energy spectra, biological model, and endpoint (Hill 2004). Correspondingly, our observation that SARRP achieved similar or better tumor control than X-Rad320 at a statistically lower measured dose underscores that physical dose alone does not dictate biological outcome. In this study, we used static beam delivery only and did not investigate the potential dosimetric advantages of conformal radiotherapy (available on some SARRP systems), which clinical experience and Monte Carlo simulations indicate can achieve superior tumor coverage and spare critical organs relative to broad‐field irradiation (Bazalova *et al* 2014). Confirming these hypotheses will require serial sectioning, spatially resolved imaging, additional biomarkers (e.g. apoptosis and vascular damage), and studies in immunocompetent models to evaluate the contribution of antitumor immunity.

Body‐weight changes were small and likely influenced by differences in anesthesia and tumor burden, so they were interpreted as a pragmatic, non‐specific indicator of well‐being rather than a definitive toxicity metric. Both platforms induced mild weight loss in most irradiated cohorts, with statistically significant reductions at 10 and 16 Gy, but the magnitude of change remained modest (within ±6% of baseline) and well below the 15% ethical threshold. Adjusting weight for tumor mass further reduced differences. No animal met humane endpoints or exhibited overt clinical toxicity. Together, these findings are consistent with the biological tolerability of fractionated 2 Gy regimens in this model. Fractionated radiotherapy exploits the greater capacity of normal tissues to repair sublethal damage between fractions (Hall and Giaccia 2011), which may have contributed to the observed tolerability in this study compared to single-shot treatments. These effects can be explored by future studies within radiobiological frameworks such as the linear quadratic model. Nevertheless, because weight change does not capture organ-specific or subclinical toxicity, future studies should integrate body‐score indices or radiation-toxicity endpoints such as diarrhea and anemia to better characterize systemic toxicity.

Normalized tumor volume AUC (nAUC) was used to evaluate tumor burden by integrating growth, suppression, and regression effects in a single metric. AUC-based approaches have been proposed previously for preclinical studies in cases where animal euthanasia or censoring produce uneven longitudinal data (Lesser *et al* 1980, Duan *et al* 2012). In this study, there was a fixed time-based endpoint, but the tumor suppression induced by therapeutic radiation doses precluded the use of conventional metrics such as tumor doubling time and growth delay. These metrics are not well defined for non-enlarging tumor behavior, as was observed in several cohorts, and their estimation would require imprecise extrapolation about tumor growth. AUC therefore provided an appropriate measure to quantify tumor volume growth and regression without requiring a specific volume threshold. While time-normalized nAUC was computed to standardize measurements across various treatment durations, our observations suggest that additional normalization for uneven initial tumor volumes may be warranted in future studies (Wu and Houghton 2010).

Both x‐ray platforms delivered clinically relevant fractionation, achieved statistically significant tumor control, and were well tolerated. With rigorous dosimetric calibration and QA, modern x-ray research irradiators are feasible tools for the study of fractionated radiotherapy treatment courses. While our findings highlight the utility of modern x‐ray platforms, several limitations temper the conclusions. Cohort sizes were small (five animals per irradiated group and two to three per *γ*‐H2AX cohort), limiting statistical power and precluding robust toxicity comparisons. Additionally, only one tumor model (22Rv1) was used, limiting generalizability. 22Rv1 was selected for its high tumor take (near 100%) and fast and consistent growth kinetics, which allowed the study to focus on reproducibility and tolerability of the x-ray irradiation regimens, but exact tumor control observations may vary with model radiosensitivity. The follow‐up period of 14 d after treatment delivery (19–24 d from treatment initiation) may underestimate late toxicities and overestimate durability of tumor control, particularly for lower doses. Furthermore, *γ*‐H2AX staining captures only one form of DNA damage, as apoptosis, senescence, vascular injury and normal‐tissue effects were not measured. This study did not evaluate immune-mediated mechanisms within the tumor microenvironment, such as tumor repolarization, which may influence therapeutic response (Amgoth *et al* 2020). Athymic mice do not recapitulate the immunological context of human radiotherapy, so immune‐mediated tumor control and toxicity may differ in immunocompetent models. These limitations highlight the need for larger cohorts, extended follow‐up, multiple mechanistic endpoints, and inclusion of other tumor and immune-competent models. Dose‐escalation or hypofractionated regimens could further clarify the upper limits of tumor control and better emulate contemporary clinical practice. Most importantly, rigorous dosimetric QA, including energy‐dependent output calibration and setup-specific dose calculations, must be integrated into preclinical irradiation studies to ensure reproducibility and comparability.

## 5. Conclusion

This study demonstrates that fractionated daily 2 Gy x‐ray regimens delivered using either a collimated cabinet X-Rad320 irradiator or an image‐guided SARRP can reduce the growth of 22Rv1 prostate‐cancer xenografts without overt clinical toxicity. Fractionated regimens were clinically well tolerated, and body-weight changes remained modest and below ethical toxicity thresholds. Irradiated cohorts showed decreased tumor burden relative to controls, but high intra-cohort variance at higher nominal doses limited statistical significance. Tumor volume curves from SARRP-treated cohorts showed apparent regression at 16 Gy and sustained suppression at 20 Gy, whereas those from X‐Rad320 exhibited delayed growth without regression. Histological analyses suggested a dose response pattern in DNA DSBs, further supporting the biological efficacy of x‐ray platforms. However, discrepancies between prescribed and absorbed doses, revealed by alanine dosimetry, underscore the critical need for platform‐specific calibration and QA. These results underscore the importance of accurate output calibration and dose planning for achieving reproducible tumor control in preclinical irradiation studies.

## Data Availability

All data that support the findings of this study are included within the article (and any supplementary information files). Supplementary information available at https://doi.org/10.1088/1361-6560/ae7953/data1.
